# The Effect on Proliferation and Differentiation of Cementoblast by Using Sclerostin as Inhibitor

**DOI:** 10.3390/ijms141021140

**Published:** 2013-10-21

**Authors:** Xingfu Bao, Yuyan Liu, Guanghong Han, Zhigang Zuo, Min Hu

**Affiliations:** 1 Department of Orthodontics, School of Stomatology, Jilin University, Changchun 130021, China; E-Mail: baoxingfu@yeah.net; 2 Department of Endodontics, School of Stomatology, Jilin University, Changchun 130021, China; E-Mails: 13756466950@163.com (Y.L.); hangh@jlu.edu.cn (G.H.); 3 Department of Orthodontics, School of Stomatology, Tianjin Medical University, Tianjin 300014, China; E-Mail: hizuo.student@sina.com

**Keywords:** sclerostin, cementoblast, root resorption, inhibitor

## Abstract

Cementogenesis is of great importance for normal teeth root development and is involved in the repair process of root resorption caused by orthodontic treatment. As highly differentiated mesenchymal cells, cementoblasts are responsible for this process under the regulation of many endogenous agents. Among these molecules, sclerostin has been much investigated recently for its distinct antagonism effect on bone metabolism. Encoded by the *sost* gene, sclerostin is expressed in osteocytes and cementocytes of cellular cementum. it is still unclear. In the current study, we investigated the effects of sclerostin on the processes of proliferation and differentiation; a series of experiments including MTT, apoptosis examination, alkaline phosphatase (ALP) activity, gene analysis, and alizarin red staining were carried out to evaluate the proliferation and differentiation of cementoblasts. Protein expression including osteoprotegerin (OPG) and receptor activator of nuclear factor kappa B ligand (RANKL) were also checked to analyze changes in osteoclastogenesis. Results show that sclerostin inhibits cementoblasts proliferation and differentiation, and promotes osteoclastogenesis. Interestingly, the monoclonal antibody for sclerostin has shown positive effects on osteoporosis, indicating that it may facilitate cementogenesis and benefit the treatment of cementum related diseases.

## 1. Introduction

As a special mineralized tissue on the surfaces of teeth roots, cementum usually connects with alveolar bone via fibers and retains the position of teeth. Consisting of 61% mineral, 27% organic matrix and 12% water, cementum has a similar composition to bone [1,2]. Being an important functional section, the organic matrix in cementum is composed of collagenous proteins, mainly type I collagen (COL I), and non-collagenous proteins such as osteocalcin (OCN), osteopontin (OPN), bone sialoprotein (BSP), osteonectin, proteoglycans and several growth factors [3]. Accord to its position and composition, cementum is divided to four different subtypes, among which acellular extrinsic fiber cementum (AEFC) and cellular intrinsic fiber cementum (CIFC) are the two most abundant types [4]. Due to the similarity between cementum and bone in composition, diseases that affect the properties of bone often have an effect on cementum as well. One of the most common such diseases is orthodontics induced root resorption, which causes the loss of root cementum even dentin [5,6]. Current understanding indicates that root resorptions have been observed in more than 90% of the orthodontic teeth [6,7]. Several previous reports have demonstrated that overloading and aseptic inflammation triggered the resorption process and the related process was administered via osteoclasts under regulation of the osteoprotegerin (OPG)/receptor activator of nuclear factor kappa B ligand (RANKL) signal [8,9]. Once the resorption process is terminated, cementoblasts begin to refill the resorption lacunae with CIFC in the majority of the patients [10,11], though approximately 3% of orthodontically treated patients fail to repair these resorptions with cementum. Thus, obvious length reduction in teeth roots and increased mobility usually exist among these patients.

Cementoblasts are highly differentiated mesenchymal cells of the periodontal ligament (PDL) that build up cementum [4]. A series of studies have disclosed the properties of cementoblasts, including expression of mineralization related genes and alkaline phosphatase (ALP) [12], induction of mineralization *in vitro* [13] and response to mechanical loading [14]. Further investigations showed that cementoblast function is affected by a number of endogenous molecules, such as bone morphogenetic protein (BMP), prostaglandin E2 (PGE2), and amelogenins [15–18]. Since cementoblast is the main cell type for root resorption repair, it is of importance and necessity to clarify the regulation mechanisms. Recently, scletrostin, a secreted glycoprotein, has attracted the interests of many researchers because of its distinct effect on bone metabolism [19,20]. Encoded by the *sost* gene, sclerostin is mainly expressed in osteocytes. Recent studies showed that sclerostin was first found from the patients of sclerosteosis and van Buchem disease, which was often associated with high bone mass [21,22]. Animal experiments based on transgenic mice with gain or loss function of *sost* have shown that slerostin can antagonise bone formation by both inhibiting bone formation and inducing bone resorption [23–25]. In addition it is now widely accepted that sclerostin is also involved in the response of bone to mechanical loading. When osteocytes sense the mechanical load, they reduce the expression of sclerostin [26,27]. This down-regulation allows more Wnts to bind with low-density lipoprotein receptor-related protein 5 (Lrp5) and activate Wnt/β-catenin signaling pathways [28]. Due to the biological actions of sclerostin, a monoclonal antibody was created and employed to treat osteoporosis [29,30].

Sclerostin is expressed in both osteocytes and cementocytes in dental tissues [31]. To the best of our knowledge, there is still no evidence to clarify if cementocytes have similar mechanical sensing ability as osteocytes do in bone. However, secreted sclerostin may penetrate through the periodontal ligament and affect the cementoblast function when orthodontic force is loaded. The effects of sclerostin on cementoblast will thus be useful to more fully understand to benefit the treatment of root resorption.

## 2. Results and Discussion

**Figure 1:**
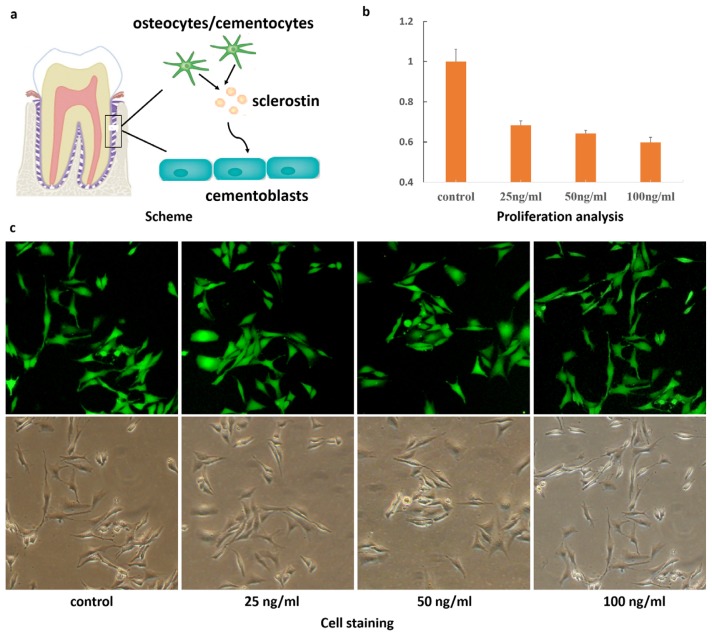
(**a**) Schematic illustration of the effect on cementoblasts through use of sclerostin secreted by osteocytes; (**b**) Proliferation analysis of cementoblasts in the presence of sclerostin; (**c**) Live-cell staining treated by sclerostin with different concentrations.

Accumulated studies showed that acellular cementum is usually seen at the beginning of repair and always covered by cellular cementum, which constitutes the main type of repaired tissue [10,11]. The cells responsible for cellular cementum formation are cementoblasts, and it is therefore important to clarify their regulatory mechanisms. It has been well accepted that cementoblasts originate from dental follicle cells in root development or periodontal cell precursors in the repair process [4]. Studies on bone metabolism concluded that sclerostin antagonises bone formation through wnt/β-catenin signaling. As a tissue lying closely to alveolar bone, little is known regarding the function of sclerostin in cementum metabolism (Figure 1a). In the present study, the effects of sclerostin on the reduction of cementoblast proliferation and differentiation were examined.

**Figure 2:**
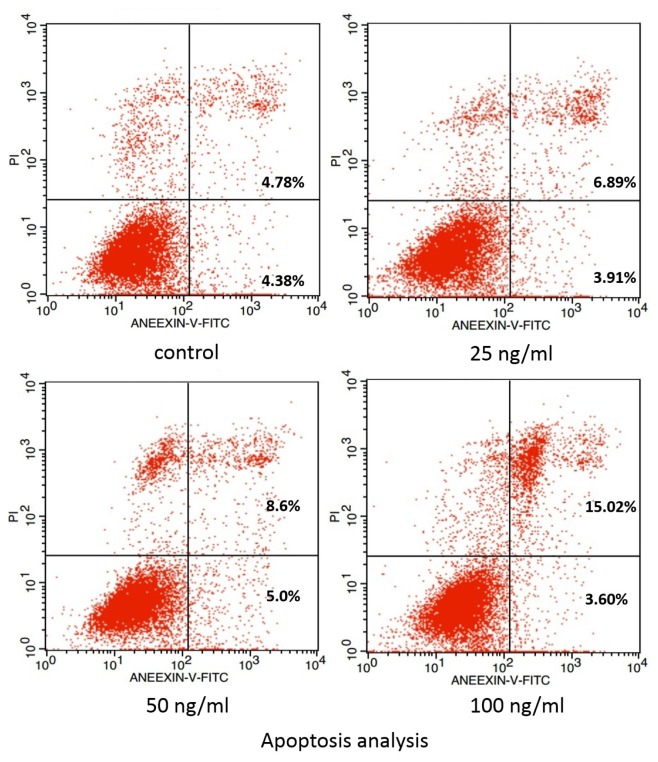
Apoptosis analysis of cementoblasts in the presence of sclerostin.

As shown in Figure 1b, fewer cells were observed in the groups treated with sclerostin compared to the control group after 24 h incubation, indicating the dose-dependent inhibitory effect. Otherwise, live-cell staining via calcein-AM illustrated in Figure 1c also demonstrated above results. Comparison of cell morphology demonstrated that there was no significant difference between control group and experimental group in the presence of various concentrations of sclerostin. It is well accepted that proliferation is an essential process for cementogenesis, especially in the repair duration of root resorption. In our study, sclerostin was shown to inhibit proliferation and impair the root resorption process. The result of apoptosis shown in Figure 2 is in compliance with our MTT and cell staining results. Specifically, cell apoptosis increased in the presence of sclerostin compared to the control group and the degree of apoptosis was in addition, highly dose-dependent, in accordance with previous reports that sclerostin promotes apoptosis of human osteoblastic cells [32]. Thereby, the effect of sclerostin on cementoblast apoptosis obtained via our model should provide new insight on cementogenesis.

**Figure 3:**
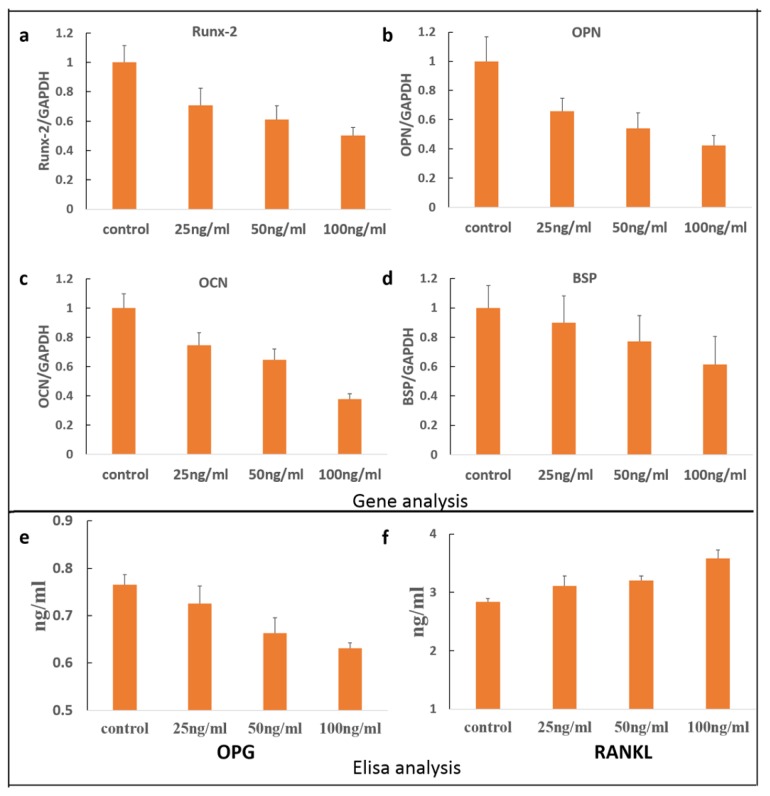
(**a**–**d**) Expression of genes related with cementoblast differentiation. Expression of OPG (**e**) and RANKL (**f**) proteins in the presence of sclerostin.

Transcripts for Runx2, OPN, OCN, and BSP in the presence of sclerostin, at concentrations of 25, 50, and 100 ng/mL, were measured after 24 h incubation. As shown in Figure 3, the presence of sclerostin significantly affects the expression of Runx2, OPN, OCN, as well as BSP. As shown in Figure 3a–d, gene expression was down-regulated in comparison to the control group. From the viewpoint of composition, cementum is approximately 45%–50% hydroxyapatite and 50% collagen and non-collagenous matrix proteins. However, non-collagenous proteins could be found in cementum including BSP, ALP, dentin matrix protein 1, fibronectin, OC, osteopontin, and several growth factors. Runx2 usually acts as a positive regulator of ALP, BSP, and OCN during the process of cementoblast differentiation. Previous research has suggested that the Wnt signaling pathway may decrease cementoblast differentiation by inhibiting Runx2 expression [33]. This is consistent with the current study that *Runx-2* gene expression was inhibited by sclerostin. OPN is a negative regulator of calcification, likely through inhibition of mineral crystal growth at a later stage of osteogenic differentiation. OCN is a late marker for cementoblast differentiation and regulates the mineral deposition. BSP, which is mainly lying on the root surface while cementogenesis occurs, has been proved to be involved in the trigger of mineralization and enhances the adhesion and differentiation of cementoblasts [34,35]. Therefore, gene analysis based on our study indicated that sclerostin may inhibit cementoblast differentiation through down regulation of mineralization related genes including Runx2, OCN, OPN, as well as BSP.

We also examined the expression of proteins related to resorbing activity including RANKL and OPG. As a critical agent on bone resorption, RANKL is well known for its distinct effect on osteoclast differentiation and action. Acting as a RANKL antagonist, OPG can inhibit its effect, and further reduce osteoclast maturation following bone resorption. As described in previous reports, cementoblasts also express RANKL and OPG as in osteoblasts [14]. It is the ratio between RANKL/OPG expression levels that controls cementoblast formation. As shown in Figure 3e,f, OPG expression induced by sclerostin is significantly down-regulated, while the expression of RANKL was significantly up-regulated. During orthodontic tooth movement, hyperactive osteoclastogenesis is believed to be a main factor during the formation of root resorption. Our findings indicated that sclerostin acted as a reinforcing agent to root cementum resorption through OPG/RANKL signaling, in accordance with a previous study [36].

**Figure 4:**
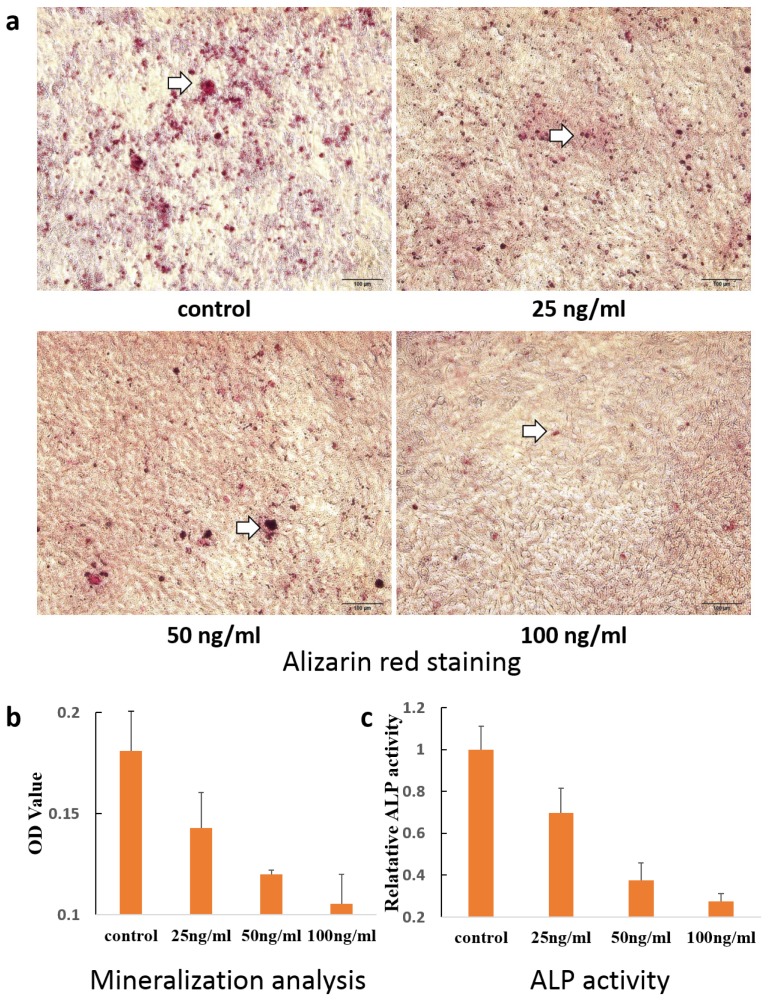
(**a**,**b**) Alizarin red staining results and quantified analysis of cementoblasts cultured with sclerostin. Inset arrow indicated the mineralization nodules; (**c**) ALP activity in the presence of sclerostin.

Classical alizarin red-S staining and OD value analysis have been used to evaluate the level of mineralization. As shown in Figure 4a, upon 8 days of co-incubation with sclerostin, red-stained mineralized nodules were clearly observed from the control group and treated group (concentrations of 0, 25, 50, 100 ng/mL). However, due to the effects of sclerostin on cementoblast differentiation, the degree of mineral nodule formation decreased with the increase of sclerostin, measured and qualified via detailed OD values as shown in Figure 4b. As an early marker of cementoblasts differentiation, ALP usually plays an important role in transferring phosphate groups from the cells to the matrix. As illustrated in Figure 4c, ALP activity of the cementoblast also decreased with the increase of sclerostin concentration in a range from 25 ng/mL to 100 ng/mL, which was highly consistent with our alizarin red-S staining results. Taken together, the presence of sclerostin resulted in a decrease of ALP activity and further induced the differentiation of cementoblasts. This is consistent with the effect of sclerostin on osteoblasts, which indicates that cementoblasts share similar characteristics with osteoblasts [37].

As a member of the DAN (differential screening–selected gene aberrant in neuroblastoma) family of glycoproteins, sclerostin has a cysteine knot structure, which is believed to antagonize bone morphogenetic protein (BMP) activity [38,39]. However, accumulated studies have suggested that sclerostin is not a classical BMP antagonist but acts through antagonizing canonical Wnt signaling in osteoblasts [40,41]. The current study has focused on the effect of sclerostin on cementoblasts proliferation and differentiation; further investigations are necessary, however, to clarify the specific mechanisms involved.

Since the discovery of sclerostin expression in osteocytes, few investigations have focused on the function of it within cementum. Expression of sclerostin protein by osteocytes in alveolar bone and cementocytes in cellular cementum was identified in both human and mouse tissues [31]. The expression of sclerostin during the cementogenesis in mice showed that sclerostin was identified only in the late stages of cementum development, and was restricted in cementocytes of cellular cementum [42]. *In vitro* experiments also indicated that *sost* mRNA was expressed only after ALP and OCN expression in the periodontal ligament cells [31]. Altered growth of cementum can be easily observed in patients with van Buchem Disease, which is due to the sclerostin abnormality [43]. Here we show that sclerostin inhibits cementoblast proliferation and differentiation, providing significant progress in the understanding of cementum metabolism. However, more studies are needed before this advance can be translated into clinical treatment applications.

## 3. Experimental Section

### 3.1. Cell Culture

An immortalized murine cementoblast cell line (OCCM-30), which was a gift from Professor Somerman at the University of Washington, was prepared as described previously. OCCM-30 cells were maintained in Dulbecco’s modified Eagle’s medium/F12 (DMEM/F12; Gibco, Carlsbad, CA, USA), supplemented with 10% fetal bovine serum (Gibco, Carlsbad, CA, USA) containing 100 U/mL penicillin and 100 μg/mL streptomycin in a humidified atmosphere of 5% CO_2_ at 37 °C. For recombinant human sclerostin (R & D Systems, Minneapolis, MN, USA) experiments, cells were treated with 25 ng/mL, 50 ng/mL and 100 ng/mL sclerostin in α-MEM supplemented with 5% fetal bovine serum.

### 3.2. MTT Assay

Cells were seeded at a density of 3 × l0^3^ per well in 96-well plates in α-MEM containing 1% FBS for 24 h, then media were changed to α-MEM with sclerostin of different concentrations. After 24 h incubation, 10 μL MTT (5 g/L) was added to each well and incubated for another 4 h. Supernatant was then removed and 150 μL DMSO was added. It was shaken for 10 min until the crystal was dissolved. The absorbance at 490 nm was measured with a micro-ELISA reader (Synergy2, Biotek, Winooski, VT, USA). The proliferation rate was calculated compared to the control group.

### 3.3. Cell Staining

Twenty thousand cells per well were cultured overnight in 24-well plates and treated with sclerostin for 24 h. Then cell morphology was assessed using calcein-AM staining. Briefly, cells in 24-well plates were washed twice with PBS (phosphate-buffered saline, pH 7.4), then cells were stained with calcein-AM (10 ng/mL) for 20 min at 4 °C in the dark. Cells were observed using fluorescence microscopy (excitation (*E*_x_), 365 nm and emission (*E*_m_), 480 nm).

### 3.4. Examination of Apoptosis

Cells were incubated with or without sclerostin for 24 h, washed twice with PBS, adjusted to 100 μL of the solution and transferred to a 1-mL centrifuge tube (2 × 10^5^ cells). Then, 5 μL of Annexin V–FITC and 10 μL of PI were added and cells were gently vortexed. Cells were then incubated for 15 min at room temperature in the dark and 400 μL of 1× binding buffer was added to each tube. Finally, cells were analyzed by flow cytometer (FACS101, Becton Dickinson, Franklin Lakes, NJ, USA).

### 3.5. Quantitative RT-PCR Analyses

Since GAPDH has been applied as the most widely used internal control gene in molecular genetic techniques, including studies focused on the OCCM-30 cementoblast lineage, we selected it as a reference gene here. Pre-experiments based on our system indicated that the *C*t values of GAPDH in different groups were very similar with each other. We thus believe that the expression of GAPDH is consistent in these circumstances, and may be utilzed as an efficient and single reference gene in our study [15,34,44].

In detail, cells were seeded in a 24-well plate at 10^4^/well to determine gene expression. After 24 h incubation, media was changed to α-MEM conditioned media containing 5% FBS, 50 mg/mL ascorbic acid and 10 mmol/L β-glycerolphosphate supplemented with sclerostin. After a subsequent 24 h culture, total RNA was isolated using EASY spin Plus (BLKW, Beijing, China) according to the manufacturer’s instructions. RNA was quantified spectrophotometrically at 260 nm (Nanodrop ND1000, Wilmington, DE, USA), and quality was examined using the ratio of absorption at 260 and 280 nm with a ratio between 1.9 and 2.1 as acceptable. Reverse transcription was performed with 2 μg of total RNA using PrimeScript Rtreagent Kit (Takara Co, Otsu, Japan). Each sample was then analyzed by quantitative real-time PCR (qPCR) (Stratagene MX3000P, La Jolla, CA, USA) in the SYBR Premix Ex TapII (Takara Co, Otsu, Japan), setting the cycles as follows: 10 s/95 °C PCR initial activation step; 40 cycles of denaturation for 20 s/95 °C and annealing step for 20 s/60 °C. Amplification efficiency of Runx-2, OPN, OCN, BSP and GAPDH is 99%, 93.4%, 99.9%, 98.6% and 100%, respectively. The change in mRNA levels was determined by the formula 2^−(ΔΔ^*^CT^*^)^, where Δ*CT* is the value from the threshold cycle (*CT*) of the treated sample subtracted from the *CT* value of untreated or zero time-point control sample. The relative amount of mRNA in the sample was normalized to GAPDH mRNA. Primers (Invitrogen, Carlsbad, CA, US) used in this research were shown in Table 1 according to previous reports.

### 3.6. OPG/RANKL Elisa Assay

Cells were seeded in 24-well plates at a concentration of 5 × 10^4^ cells/well, and cultured for 24 h. Then the culture medium was changed to alpha-MEM with or without sclerostin. Culture medium was collected after another 24 h culture and prepared for OPG and RANKL ELISA assay. The assays were performed according to the protocol of the manufacturer using a specific ELISA kit (BlueGene Biotech, Shanghai, China). Each protein sample was analyzedin triplicate with parallel 3-well culture plates to ensure accurate results.

### 3.7. Alizarin Red Staining

Cells were plated in 24-well plates in DMEM/F12 containing 10% FBS. After 24 h, cells were changed to α-MEM conditioned media containing 5% FBS, 50 mg/mL ascorbic acid and 10 mmol/L β-glycerolphosphate, plus different concentrations of sclerostin. The media was changed every 4 days and mineralization of extracellular matrix was evaluated on day 10 by Alizarin red staining. After taking photographs, 1mL cetylpyridinimu chloride (1%) was added into the plate, followed by measuring OD values at spectrophotometrically at 540 nm.

### 3.8. Alkaline Phosphatase Activity

Cells seeded in 24-well plates were incubated with α-MEM conditioned media containing 5% FBS, 50 mg/mL ascorbic acid and 10 mmol/L β-glycerolphosphate supplemented with sclerostin. Media was changed every 3 days and cells were dissolved in 0.05% Triton X-100 buffer 7 days later. Cell lysates were analyzed for ALP activity in 96-wells plates according to instructions. The plates were then read spectrophotometrically at 405 nm. All ALP activity results were normalized to the total protein in the cell lysate.

### 3.9. Statistical Analysis

All experiments in this study were performed three times, and representative findings are shown. Experimental values are given as means ± SD. One-way analysis of variance (ANOVA) was carried to determine the significance between control and treatments groups.

## 4. Conclusions

Sclerostin was shown to suppress cementoblast proliferation and promote apoptosis, while differentiation was also inhibited in a dose-dependent manner. Osteoclastogenesis affected by OPG and RANKL was enhanced in the presence of sclerostin. These findings along with previous studies indicate that sclerostin is involved in homeostasis through the negative regulation of cellular cementum formation. Sclerostin thereby shows significant potential as a specific target for the treatment of cementum related diseases.

## Figures and Tables

**Table 1:** Primers used for quantitative real-time PCR.

Primers	Forward	Reverse
Runx-2	CTTCATTCGCCTCACAAAC	CTAGCAGTGACGGTCT
BSP	GAGACGGCGATAGTTCC	AGTGCCGCTAACTCAA
OCN	TGAACAGACTCCGGCG	GATACCGTAGATGCGTTTG
OPN	TTTACCAGCCTGCACCC	CTAGCAGTGACGGTCT
GAPDH	ACCACAGTCCATGCCATCAC	TCCACCACCCTGTTGCTGTA
