# Xingfu Bao et al. The Effect on Proliferation and Differentiation of Cementoblast by Using Sclerostin as Inhibitor. *Int. J. Mol. Sci.* 2013, *14*, 21140

**DOI:** 10.3390/ijms20102562

**Published:** 2019-05-24

**Authors:** Xingfu Bao, Yuyan Liu, Guanghong Han, Zhigang Zuo, Min Hu

**Affiliations:** 1 Department of Orthodontics, School of Stomatology, Jilin University, Changchun 130021, China; baoxingfu@yeah.net; 2 Department of Endodontics, School of Stomatology, Jilin University, Changchun 130021, China; 13756466950@163.com (Y.L.); hangh@jlu.edu.cn (G.H.); 3 Department of Orthodontics, School of Stomatology, Tianjin Medical University, Tianjin 300014, China; hizuo.student@sina.com

The authors wish to make the following correction to this paper [1]. Due to mislabeling, replace Figure 1 with:

The change does not affect the scientific results. We apologize for the mistake.

## Figures and Tables

**Figure 1:**
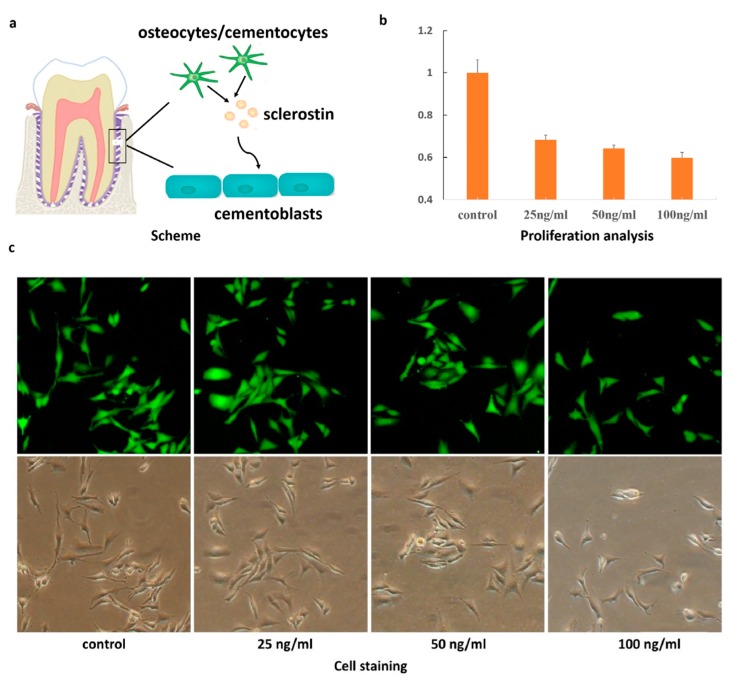
(**a**) Schematic illustration of the effect on cementoblasts through use of sclerostin secreted by osteocytes; (**b**) Proliferation analysis of cementoblasts in the presence of sclerostin; (**c**) Live-cell staining treated by sclerostin with different concentrations.
